# Construction of diagnostic models for the progression of hepatocellular carcinoma using machine learning

**DOI:** 10.3389/fonc.2024.1401496

**Published:** 2024-05-15

**Authors:** Xin Jiang, Ruilong Zhou, Fengle Jiang, Yanan Yan, Zheting Zhang, Jianmin Wang

**Affiliations:** ^1^ Innovation Center for Cancer Research, Clinical Oncology School of Fujian Medical University, Fujian Cancer Hospital, Fuzhou, China; ^2^ Fujian Key Laboratory of Advanced Technology for Cancer Screening and Early Diagnosis, Fuzhou, China; ^3^ Faculty of Medicine, The Chinese University of Hong Kong, Hong Kong, Hong Kong SAR, China

**Keywords:** liver cancer, machine learning, random forest model, LightGBM model, the progression of HCC

## Abstract

Liver cancer is one of the most prevalent forms of cancer worldwide. A significant proportion of patients with hepatocellular carcinoma (HCC) are diagnosed at advanced stages, leading to unfavorable treatment outcomes. Generally, the development of HCC occurs in distinct stages. However, the diagnostic and intervention markers for each stage remain unclear. Therefore, there is an urgent need to explore precise grading methods for HCC. Machine learning has emerged as an effective technique for studying precise tumor diagnosis. In this research, we employed random forest and LightGBM machine learning algorithms for the first time to construct diagnostic models for HCC at various stages of progression. We categorized 118 samples from GSE114564 into three groups: normal liver, precancerous lesion (including chronic hepatitis, liver cirrhosis, dysplastic nodule), and HCC (including early stage HCC and advanced HCC). The LightGBM model exhibited outstanding performance (accuracy = 0.96, precision = 0.96, recall = 0.96, F1-score = 0.95). Similarly, the random forest model also demonstrated good performance (accuracy = 0.83, precision = 0.83, recall = 0.83, F1-score = 0.83). When the progression of HCC was categorized into the most refined six stages: normal liver, chronic hepatitis, liver cirrhosis, dysplastic nodule, early stage HCC, and advanced HCC, the diagnostic model still exhibited high efficacy. Among them, the LightGBM model exhibited good performance (accuracy = 0.71, precision = 0.71, recall = 0.71, F1-score = 0.72). Also, performance of the LightGBM model was superior to that of the random forest model. Overall, we have constructed a diagnostic model for the progression of HCC and identified potential diagnostic characteristic gene for the progression of HCC.

## Introduction

According to the recent data on global cancer burden in 2020, liver cancer ranked as the sixth most common cancer in terms of incidence rate and the third highest in terms of mortality (1). A considerable percentage of patients diagnosed with hepatocellular carcinoma (HCC) are at an advanced stage. Therefore, the identification of diagnostic markers is of immense importance (2–4). The development of HCC is a gradual process. Patients with chronic liver disease experience persistent liver inflammation, fibrosis, and abnormal regeneration of liver cells. These abnormalities can lead to cirrhosis and gradually give rise to dysplastic nodules of precancerous lesions. Finally, the patients will develop HCC (5). However, the marker gene for HCC progression remain unclear.

Thus, there is an urgent need to identify markers and develop precise diagnostic model for progression of HCC. With the development of artificial intelligence, machine learning has shown promise in cancer diagnosis and treatment (6, 7). For example, Zhang (8) developed a machine learning-based model for the early detection of liver cancer by utilizing low-depth whole genome sequencing of cell-free DNA. The model achieved an AUC of 0.995, a sensitivity of 0.968, and a specificity of 0.988 in differentiating between liver cancer and non-liver cancer. According to feature selection, Tang (9) used Least Absolute Shrinkage and Selector Operation (Lasso), Support Vector Machine (SVM), and Random Forest (RF) to construct HCC classification models for HCC saliva samples. The diagnostic accuracy of the LASSO-HCC model was 0.706, the diagnostic accuracy of the SVM-HCC model was 0.812, and the diagnostic accuracy of the RF-HCC model was 0.859.

However, these studies exclusively focused on particular stages in the progression of HCC. In this research, we aim to develop an accurate diagnostic model for the progression of HCC by utilizing machine learning algorithms, such as RF and LightGBM. The RF and LightGBM models are two commonly used machine learning algorithms known for their strong performance and effectiveness in dealing with classification and regression problems.

RF is an ensemble learning algorithm that enhances prediction accuracy by constructing multiple decision trees and taking the average of the predictions from these trees. RF can reduce overfitting, is tolerant to missing values, and can assess the importance of each feature, aiding in data comprehension (10, 11). LightGBM is a distributed and high-performance algorithm designed for gradient-boosting decision trees, specifically based on the Histogram algorithm, characterized by efficiency, speed, and high accuracy. Principle of LightGBM is to iteratively train multiple decision trees and train the next tree based on the results of the previous tree to minimize the loss function (12, 13). Combining the RF and LightGBM models can yield more comprehensive and accurate results in research. These two algorithms have outstanding performance in cancer diagnostics (14).

In this research, we classified 118 samples from GSE114564 into three groups: normal liver, precancerous lesion, and HCC. The RF model and LightGBM model showed strong performance and identified 12 characteristic genes. Additionally, the diagnostic model still exhibited high efficacy when categorizing the progression of HCC into six finely stratified stages. To the best of our knowledge, this research represented the first application of machine learning to comprehensively cover all stages of HCC progression.

## Materials and methods

### Patients

This research employed the RNA-sequencing dataset GSE114564 (15), retrieved from the GEO database, which included transcriptome data from 118 tissue samples representing different stages of HCC. The dataset included 15 normal liver samples, 20 chronic hepatitis samples, 10 liver cirrhosis samples, 10 dysplastic nodule samples, 18 early stage HCC samples, and 45 advanced HCC samples. This comprehensive dataset covers almost all stages for progression of HCC.

### Data processing

We obtained the file “GSE114564_Liver_Cancer_FPKM.txt.gz” from the GEO database (https://www.ncbi.nlm.nih.gov/geo/query/acc.cgi?acc=GSE114564). FPKM (fragments per kilobase of exon model per million mapped fragments) of 118 samples were used as the input file, which can effectively eliminate the impact of sequencing depth and gene length on the results. Following that, we conducted an 8:2 random split (16–18) to partition the 118 available samples into training and validation sets. The 8:2 ratio is commonly regarded as a reasonable choice, because it ensures an adequate sample size for the training set, while also providing a certain number of samples for the validation set to evaluate model performance. Next, we kept genes that are expressed (FPKM>0) in at least three samples and these genes are in scanpy (19) (scanpy.pp.filter_genes). Then, the data matrix is log-transformed (scanpy.pp.log1p). In the end, we selected the top 1000 genes (20–22) by the ranking variances of all samples (scanpy.pp.highly_variable_genes), which was performed variance calculation in Scanpy. More specifically, a normalized variance for each gene is computed. First, the data are standardized (i.e., *z*-score normalization per feature) with a regularized standard deviation. Next, the normalized variance is computed as the variance of each gene after the transformation. Genes are ranked by the normalized variance. Finally, we selected the top 1,000 genes (**Supplementary Table S1**) that demonstrated the highest overall variance in FPKM as the foundation for constructing RF and LightGBM models. The variance calculation and above data processing steps were all implemented in scanpy.

### Construction of machine learning

Subsequently, we employed the Python framework sklearn (23) to construct the RF model using the RF program (sklearn.ensemble.RandomForestClassifier) and LightGBM program (Lightgbm.sklearn), with all parameters set to default values. The framework sklearn available online is: https://scikit-learn.org/stable/supervised_learning.html. Cross-validation was used in this study to find the optimal parameters of the classification model and help the model alleviate overfitting. This study uses fivefold cross-validation on the training dataset, and uses accuracy, precision, recall, and F1-score to evaluate the model performance, and the results are in **Supplementary Table S2**.

### Analysis of characteristic gene

**Figure 1:**
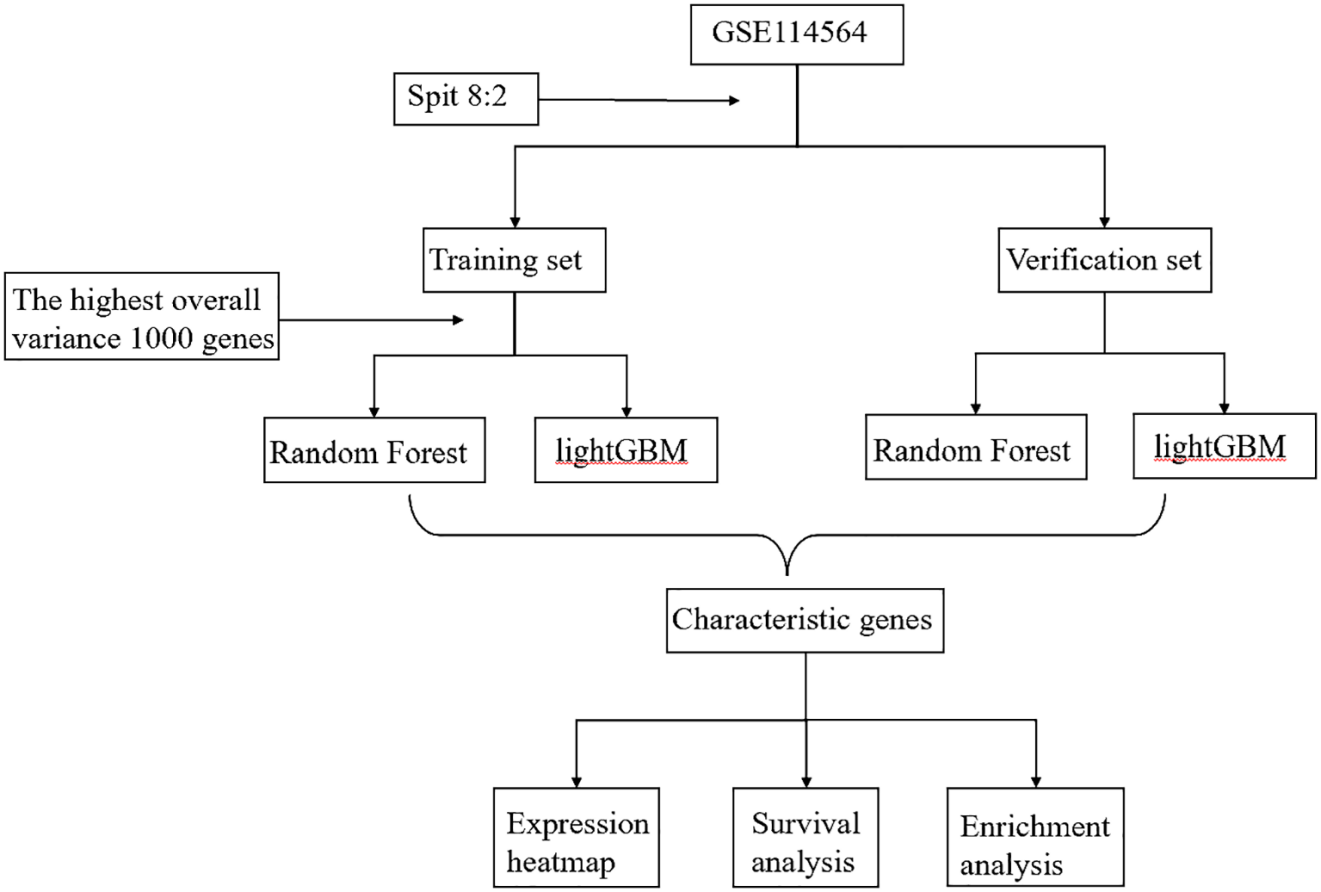
Workflow diagram in this research.

The RF and LightGBM models calculated the gene importance and identified the top 50 most important genes (24), separately (**Supplementary Table S3**). Furthermore, the intersection of these 50 genes was taken to obtain the feature genes. Upon constructing the aforementioned model, we obtained a set of characteristic genes. Following that, we generated expression heatmap using TBtools HeatMap illustrator program. TBtools is an integrative toolkit developed for interactive analyses of big biological data (25, 26). Survival analysis was performed using the GEPIA2 database, and GO pathway enrichment was performed using clusterProfiler R package (27, 28). Finally, we used the GeneCards database (29) to identify characteristic genes associated with occurrence of HCC (https://www.genecards.org/). The workflow diagram for this research was depicted in **Figure 1**.

## Result

### Constructing machine learning model based three distinct groups

**Figure 2:**
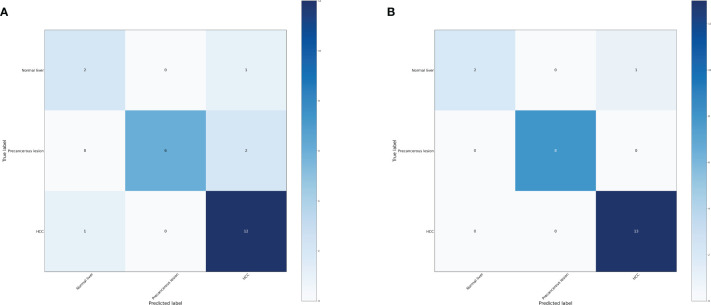
Confusion matrix of the models. **(A)** Confusion matrix of the random forest model. **(B)** Confusion matrix of the LightGBM model.

Based on the transformative process of HCC, the data can be categorized into three groups: normal liver, precancerous lesion (including chronic hepatitis, liver cirrhosis, dysplastic nodule), and HCC (including early stage HCC and advanced HCC). We employed the RF and LightGBM algorithms of machine learning to develop a diagnostic model for the progression of HCC. Performance measure of the RF model was presented in **Figure 2** and **Table 1**, indicating an accuracy of 0.83, precision of 0.83, recall of 0.83, and F1-score of 0.83. Similarly, performance measure of the LightGBM model indicated an accuracy of 0.96, precision of 0.96, recall of 0.96, and F1-score of 0.95.

**Table 1:** Performance measure of machine learning models based three distinct groups.

Model	Accuracy	Precision	Recall	F1-score
Random forest	0.83	0.83	0.83	0.83
LightGBM	0.96	0.96	0.96	0.95

**Figure 3:**
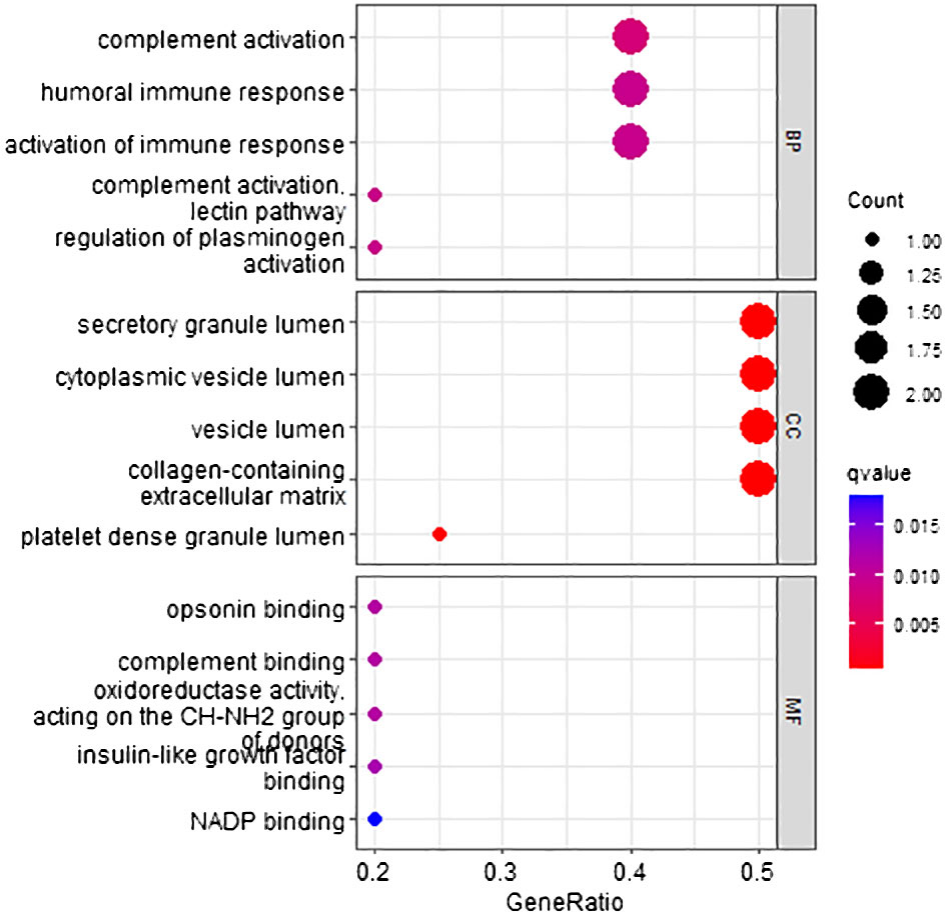
Go pathway enrichment of characteristic genes.

According to the method, the models above comprised a total of 12 characteristic genes (*CLEC3B*, *RN7SL5P*, *RP11–977G19.10*, *ASPDH*, *CFP*, *CDC37L1-AS1*, *RN7SL752P*, *U3*, *IGFALS*, *MASP2*, *RN7SKP255*, *RP11–162P23.2*). Next, we utilized TBtools to generate expression heatmap for these 12 characteristic genes (**Supplementary Figure S1**). The characteristic genes are primarily involved in complement activation, activation of immune response, cytoplasmic vesicle lumen, complement binding, oxidoreductase activity, and other pathways (*q* < 0.05; **Figure 3**).

**Figure 4:**
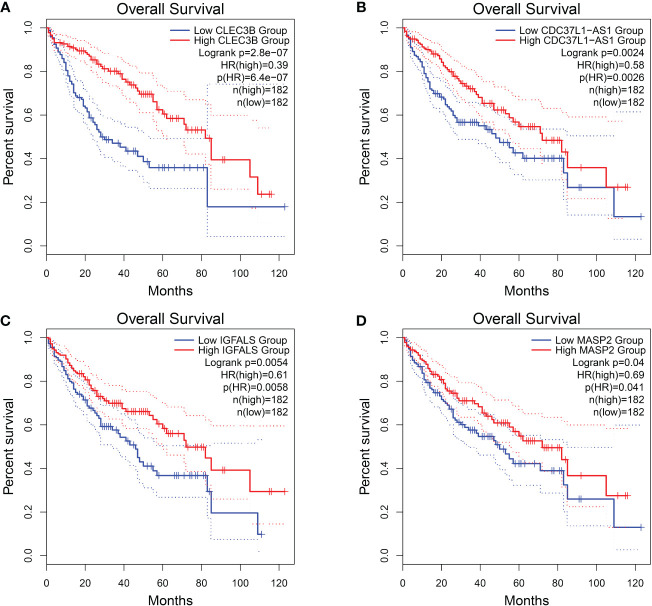
Overall survival of HCC genes in GEPIA2 database. **(A)** Overall survival of *CLEC3B.* **(B)** Overall survival of *CDC37L1-AS1.* **(C)** Overall survival of *IGFALS.* **(D)** Overall survival of *MASP2*.

Among these genes, we found that poor prognosis was associated with low expression of *CLEC3B*, *CDC37L1-AS1*, *IGFALS*, and *MASP2* (Logrank *p* < 0.05; **Figure 4**). Moreover, both *CLEC3B* and *IGFALS* showed a strong association with the occurrence of HCC (**Table 2**) (30, 31).

**Table 2:** *CLEC3B* and *IGFALS* reported in HCC from GeneCards database.

GeneName	Location	Function summaries	Related pathways	Report
*CLEC3B*	3p21.31	May be involved in the packaging of molecules destined for exocytosis.	Platelet activation, signaling and aggregation.	(30)
*IGFALS*	16p13.3	Encoded by this gene is a serum protein that binds insulin-like growth factors, increasing their half-life and the vascular localization.	1.Regulation of Insulin-like Growth Factor. 2.Inulin-like growth factor binding.	(31)

### Constructing machine learning model based four distinct groups

**Figure 5:**
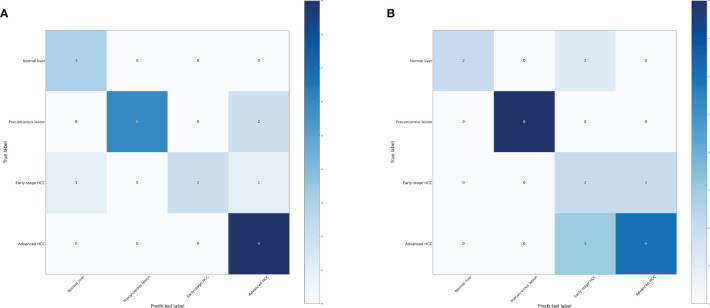
Confusion matrix of the models. **(A)** Confusion matrix of the random forest model. **(B)** Confusion matrix of the LightGBM model.

In order to further investigate the effectiveness of machine learning models in classifying early stage HCC, we categorized the data into four groups: normal liver, precancerous lesion (including chronic hepatitis, liver cirrhosis, dysplastic nodule), early stage HCC, and advanced HCC. Performance measure of the random forest model was presented in **Figure 5**, **Table 3**, indicating an accuracy of 0.83, precision of 0.83, recall of 0.83, and F1-score of 0.83. Similarly, performance measure of the LightGBM model indicated an accuracy of 0.75, precision of 0.75, recall of 0.75, and F1-score of 0.76.

**Table 3:** Performance measure of machine learning models based four distinct groups.

Model	Accuracy	Precision	Recall	F1-score
Random forest	0.83	0.83	0.83	0.83
LightGBM	0.75	0.75	0.75	0.76

**Figure 6:**
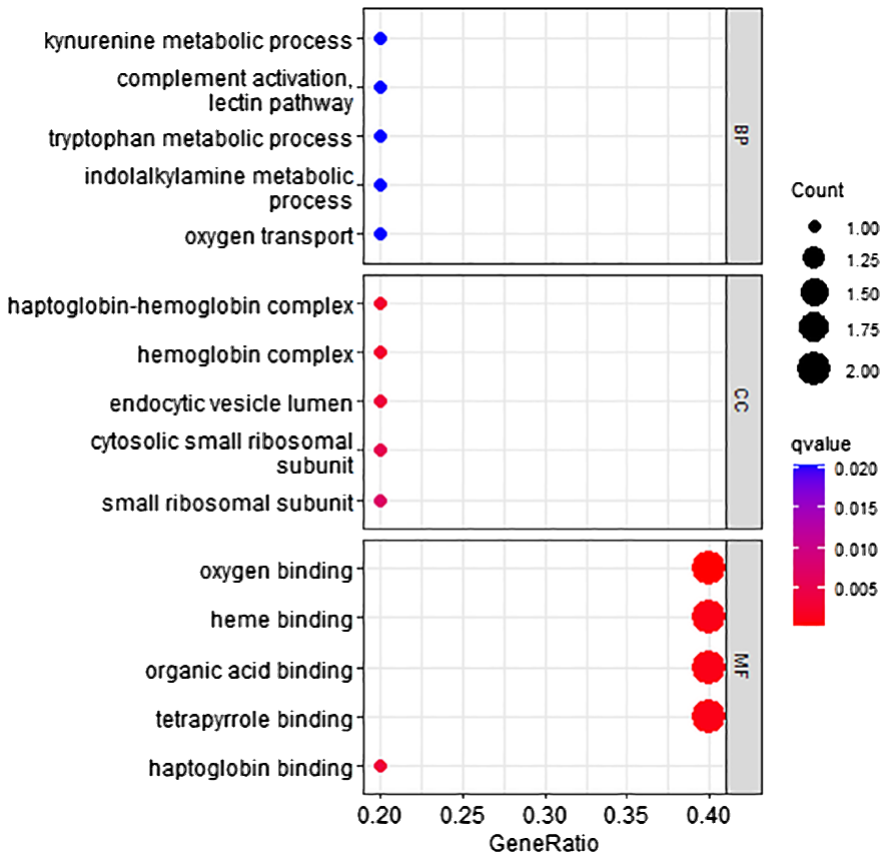
GO pathway enrichment of characteristic genes.

According to the method, the models above comprised a total of 12 characteristic genes (*HBA2*, *RP11–977G19.10*, *AC004538.3*, *INS-IGF2*, *RNU2–63P*, *RN7SL752P*, *U3*, *VIPR1*, *MASP2*, *TDO2*, *RN7SKP255*, *RP11–162P23.2*). Furthermore, we utilized TBtools to generate expression heatmap for these 12 characteristic genes (**Supplementary Figure S2**). The characteristic genes are primarily enriched in pathways associated with the tryptophan metabolic process, hemoglobin complex, oxygen binding, and other pathways (*q* < 0.05; **Figure 6**).

**Figure 7:**
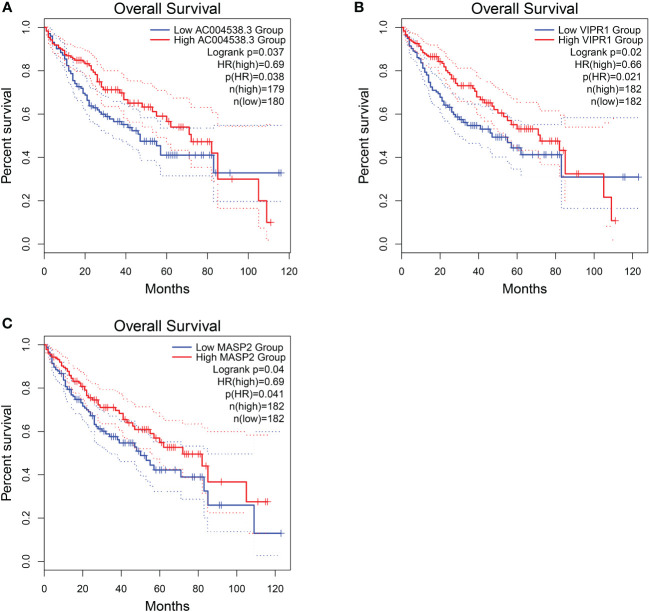
Overall survival of HCC genes in GEPIA2 database. **(A)** Overall survival of *AC004538.3*. **(B)** Overall survival of *VIPR1*. **(C)** Overall survival of *MASP2.*.

Regarding these genes, low expression of *AC004538.3*, *VIPR1*, and *MASP2* was associated with a poor prognosis (Logrank *p* < 0.05; **Figure 7**). Furthermore, *VIPR1* exhibited a strong association with the occurrence of HCC (**Table 4**) (32).

**Table 4:** *VIPR1* reported in HCC from GeneCards database.

GeneName	Location	Function summaries	Related pathways	Report
*VIPR1*	3p22.1	This is a receptor for VIP. The activity of this receptor is mediated by G proteins which activate adenylyl cyclase.	1.Glucocorticoid receptor regulatory network. 2.GPCR downstream signal.	(32)

### Constructing machine learning model based six distinct groups

**Figure 8:**
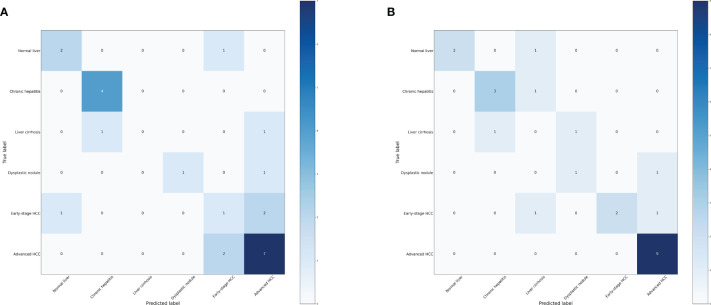
Confusion matrix of the models. **(A)** Confusion matrix of the random forest model. **(B)** Confusion matrix of the LightGBM model.

We further investigated the efficacy of classifying the progression of HCC across all various stages. To achieve this, we categorized the data into six groups: normal liver, chronic hepatitis, liver cirrhosis, dysplastic nodule, early stage HCC, and advanced HCC. Performance measure of the random forest model was presented in **Figure 8** and **Table 5**, indicating an accuracy of 0.63, precision of 0.63, recall of 0.63, and F1-score of 0.59. Similarly, performance measure of the LightGBM model indicated an accuracy of 0.71, precision of 0.71, recall of 0.71, and F1-score of 0.72.

**Table 5:** Performance measure of machine learning models based six distinct groups.

Model	Accuracy	Precision	Recall	F1-score
Random forest	0.63	0.63	0.63	0.59
LightGBM	0.71	0.71	0.71	0.72

**Figure 9:**
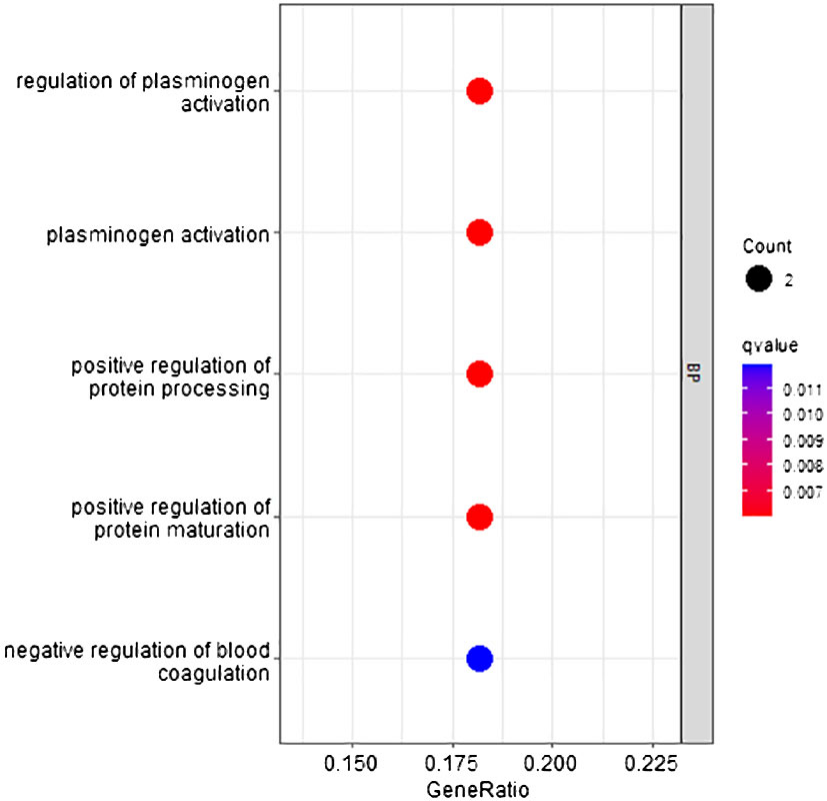
GO pathway enrichment of characteristic genes.

According to the method, the models above comprised a total of 16 characteristic genes (*C1QTNF1*, *JUNB*, *CLEC3B*, *SERPINA11*, *RP11–977G19.10*, *CCNB1*, *CDC37L1-AS1*, *CFB*, *RN7SL752P*, *CCL14*, *U3*, *F12*, *ACSL4*, *MOGAT2*, *RN7SKP255*, and *TERC*). Furthermore, we utilized TBtools to generate expression heatmap for these 16 characteristic genes (**Supplementary Figure S3**). The characteristic genes are primarily enriched in pathways associated with regulation of plasminogen activation, positive regulation of protein processing, and other pathways (*q* < 0.05; **Figure 9**).

**Figure 10:**
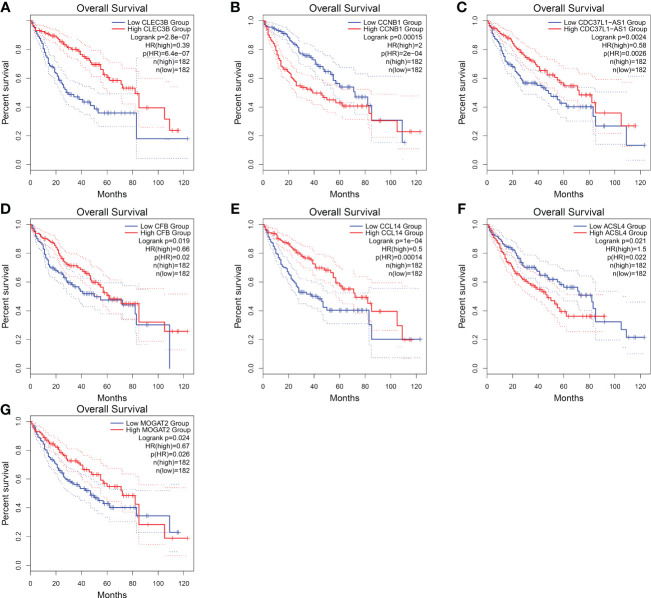
Overall survival of HCC genes in GEPIA2 database. **(A)** Overall survival of *CLEC3B.* **(B)** Overall survival of *CCNB1*. **(C)** Overall survival of *CDC37L1-AS1*. **(D)** Overall survival of *CFB*. **(E)** Overall survival of *CCL14.* **(F)** Overall survival of *ACSL4*. **(G)** Overall survival of *MOGAT2*.

Regarding these genes, low expression of *CLEC3B*, *CDC37L1-AS1*, *CFB, CCL14*, and *MOGAT2* was associated with poor prognosis, while high expression of *CCNB1* and *ACSL4* was associated with a poor prognosis (**Figure 10**). Furthermore, *CLEC3B*, *CCNB1*, *CCL14*, and *ACSL4* exhibited a strong association with the occurrence of HCC (**Table 6**) (30, 33–35).

**Table 6:** *CLEC3B*, *CCNB1*, *CCL14*, and *ACSL4* reported in HCC from GeneCards database.

GeneName	Location	Function summaries	Related pathways	Report
*CLEC3B*	3p21.31	Tetranectin binds to plasminogen and to isolated kringle 4. May be involved in the packaging of molecules destined for exocytosis.	Platelet activation, signaling and aggregation.	(30)
*CCNB1*	5q13.2	Essential for the control of the cell cycle at the G2/M (mitosis) transition.	1.AMPK signaling pathway. 2. Cell cycle	(33)
*CCL14*	17q12	This gene, chemokine (C-C motif) ligand 14, is one of several CC cytokine genes clustered.	1.MIF-mediated glucocorticoid regulation and TGF-Beta Pathway. 2.Chemokine activity.	(34)
*ACSL4*	Xq23	Catalyzes the conversion of long-chain fatty acids to their active form acyl-CoA for both synthesis of cellular lipids.	Fatty acid metabolism.	(35)

## Discussion

In this research, we employed machine learning algorithms, specifically random forest and LightGBM, to develop accurate diagnostic models for progression of HCC. After multiple analyses, we have identified potential diagnostic markers for the progression of HCC. Interestingly, when we categorized samples into three groups, the classification accuracy of LightGBM algorithm exceeded 0.95. Also, performance of the random forest model was slightly inferior compared to the LightGBM model. The 12 characteristic genes are primarily involved in complement activation, activation of immune response pathways. Simultaneously, among the characteristic gene *CLEC3B* generated from the model, exosomes derived from HCC with downregulated *CLEC3B* were found to promote the migration, invasion, and epithelial-mesenchymal transition of both tumor cells and endothelial cells (30). In addition, the *IGFALS*, a tumor suppressor gene, undergoes epigenetic silencing, leading to dysregulation of the IGF-II signaling in HCC (31). Our research indicated that the *CLEC3B* and *IGFALS* may be involved in the progression from normal liver to precancerous lesions to HCC, but their functions require further investigation.

Furthermore, we explored whether this model can accurately distinguish early stage HCC and assessed the potential benefits of early stage HCC diagnosis. And when the samples were categorized into four groups, the random forest model achieved a classification accuracy exceeding 0.83. Moreover, performance of the LightGBM model was slightly inferior compared to the random forest model. The 12 characteristic genes are primarily enriched in pathway associated with metabolic process. Among the characteristic gene generated from the model, loss of *VIPR1* expression in HCC facilitated CAD phosphorylation and tumor progression, suggesting that the restoration of *VIPR1* and treatment with the *VIPR1* agonist may represent a promising approach for HCC treatment (32, 36). Our research suggested that *VIPR1* may play a role in the classification of early stage HCC and advanced HCC, but further research is needed to determine its specific function.

Moreover, when categorizing the stages of HCC into six distinct levels, the model still exhibits high diagnostic efficacy. These findings provide a solid foundation for precise treatment. The 16 characteristic genes are primarily enriched in pathway associated with positive regulation of protein processing. Among the characteristic gene generated from the model, *CCNB1* may participate in the cell cycle of HCC by regulating DNA replication, thus promoting the development of HCC (33). And, *CCL14* was a potential prognostic biomarker for determining HCC progression and was associated with immune cell infiltration in HCC (34, 37). *ACSL4* promoted the progression of HCC by stabilizing c-Myc through the ERK/FBW7/c-Myc axis (38). Our research suggested that these genes may be involved in all stages of HCC progression and serve as potential biomarkers. However, further in-depth research is needed.

In the past 20 years, sequencing technologies have continuously advanced, leading to explosive growth in available data. Artificial intelligence is often used for the characterization of sequencing data, which can enhance the ability to detect HCC tumors and provide information for disease diagnosis and staging (39).

Xie (40) utilized gene expression profiles from peripheral blood to develop an artificial neural network (ANN) model that could differentiate HCC patients from the control group with a sensitivity of 96% and specificity of 86%. Harpreet (41) utilized a large-scale transcriptomic analysis dataset containing a total of 2,316 HCC samples and 1,665 non-tumor tissue samples to identify HCC samples using machine learning, with an accuracy ranging from 93% to 98%. Although these studies have demonstrated good predictive performance, they did not further differentiate and study non-tumor tissues (pre-cancerous stages).

In addition, A single-center prospective study in the UK recruited 331 cases of liver cell carcinoma, with a control group involving only 339 patients with chronic liver disease. A logistic regression analysis model was constructed, with an AUROC of 0.97 indicating excellent predictive performance. However, the study was only validated in a cohort of patients with fatty liver disease (42). Xing (43) conducted mass spectrometry proteomics sequencing and built a random forest machine learning model that clearly distinguished between HCC and healthy individuals (sensitivity 0.975, specificity 1.000), as well as between HCC and cirrhosis (sensitivity 0.925, specificity 0.915). However, these studies did not cover all stages of liver cancer progression.

In our study, we comprehensively cover all stages of liver cancer development, including normal liver, chronic hepatitis, liver cirrhosis, dysplastic nodule, early stage HCC, and advanced HCC. Furthermore, we conducted detailed classifications into three categories, four categories, and six categories respectively, in order to systematically study relevant models of liver cancer progression. When we categorized three groups: normal liver, precancerous lesion (including chronic hepatitis, liver cirrhosis, dysplastic nodule) and HCC (including early stage HCC and advanced HCC), The LightGBM model exhibited outstanding performance (accuracy = 0.96, precision = 0.96, recall = 0.96, F1-score = 0.95). Surprisingly, when the progression of HCC was categorized into the most refined six stages, the diagnostic model still demonstrated high performance (accuracy = 0.71, precision = 0.71, recall = 0.71, F1 score = 0.72). In conclusion, we successfully constructed the most detailed model of HCC progression stages using machine learning methods, providing a theoretical basis for accurate diagnosis of HCC.

In summary, this research represented the pioneering construction of a diagnostic model for HCC progression through the utilization of machine learning methods. The development of liver cancer is a gradual process. Liver cancer patients undergo a process from hepatitis and liver fibrosis to abnormal nodules, ultimately developing into liver cancer. By subdividing into different stages, we can more finely assess the disease progression stage of liver cancer patients and intervene with precision medicine. We hope that targeted early intervention and treatment can prevent the progression of HCC to advanced stage in the future. Additionally, we have identified key genes associated with the progression of liver cancer. Further research on these genes will facilitate the development of effective targets for liver cancer progression. It is important to note that the HCC progression characteristic genes identified in our research still lack sufficient research concerning their impact on progression of HCC, and further exploration is warranted. Of course, it is crucial to validate effectiveness of the model using a larger sample size. Due to the reduced cost of transcriptome sequencing, increasing dataset will arise in the future. In a word, this research holds potential for clinical application due to its significance and prospect.

## Data availability statement

The original contributions presented in the study are included in the article/**Supplementary Material**. Further inquiries can be directed to the corresponding author.

## Author contributions

XJ: Data curation, Formal analysis, Writing – original draft, Writing – review & editing. RZ: Data curation, Formal analysis, Software, Writing – original draft. FJ: Data curation, Investigation, Writing – review & editing. YY: Conceptualization, Investigation, Writing – review & editing. ZZ: Conceptualization, Supervision, Writing – original draft. JW: Conceptualization, Funding acquisition, Investigation, Writing – review & editing, Writing – original draft.
