# Supplementary material for: Construction of diagnostic models for the progression of hepatocellular carcinoma using machine learning
Source: Front Oncol. 2024 May 15;14:1401496. doi: 10.3389/fonc.2024.1401496 (PMC11133637; doi:10.3389/fonc.2024.1401496)
Supplement: Supplementary file 1 [file DataSheet_1.pdf]

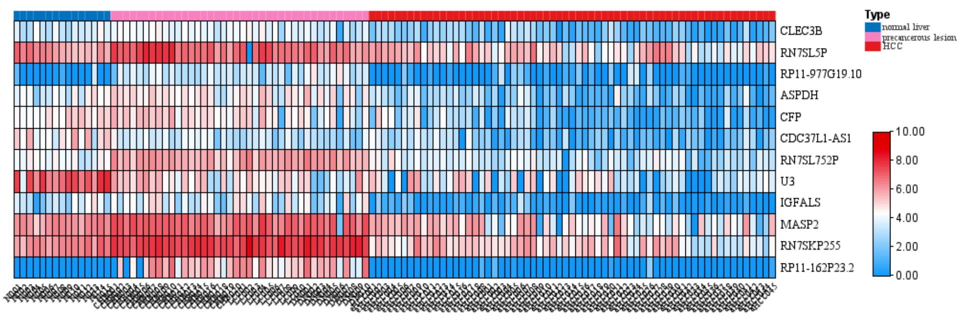

Figure S1

Utilized TBtools to generate expression heatmaps for these 12 characteristic genes in three distinct groups.
