# Supplementary material for: Construction of diagnostic models for the progression of hepatocellular carcinoma using machine learning
Source: Front Oncol. 2024 May 15;14:1401496. doi: 10.3389/fonc.2024.1401496 (PMC11133637; doi:10.3389/fonc.2024.1401496)
Supplement: Supplementary file 3 [file DataSheet_3.pdf]

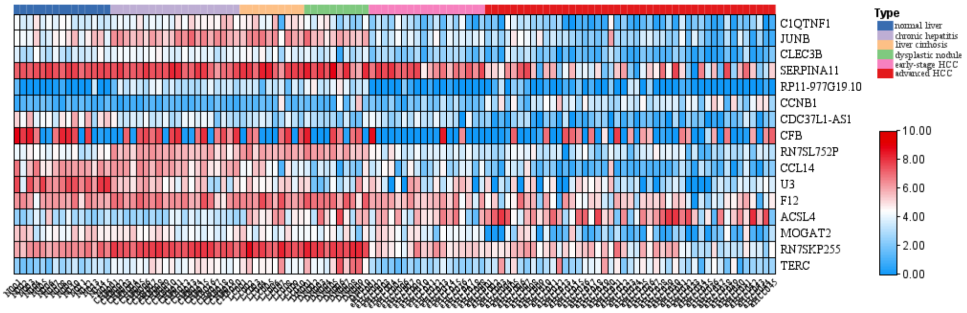

Figure S3

Utilized TBtools to generate expression heatmaps for these 16 characteristic genes in six distinct groups.
